# Supplementary material for: Effect of soybean and seaweed-based diets on growth performance, feed utilization, and gut microbiota of tilapia: A systematic review and meta-analysis
Source: PLoS One. 2024 Jul 24;19(7):e0293775. doi: 10.1371/journal.pone.0293775 (PMC11268637; doi:10.1371/journal.pone.0293775)
Supplement: S2 Table — (DOCX) [file pone.0293775.s002.docx]

**Effect of Soybean and Seaweed-based Diets on Growth Performance, Feed Utilization, and Gut Microbiota of Tilapia: A Systematic Review and Meta-analysis**

Leonildo dos Anjo Viagem^1,2,3*^; Jean Nepomuscene Hakizimana^1^; Cyrus Rumisha^2^; Brunno da Silva Cerozi^4^ and Gerald Misinzo^1,5*^

^1^ SACIDS Africa Centre of Excellence for Infectious Diseases, SACIDS Foundation for One Health, Sokoine University of Agriculture, PO Box 3297, Morogoro, Tanzania

^2^ Department of Animal, Aquaculture and Range Sciences, College of Agriculture, Sokoine University of Agriculture, PO Box 3004, Morogoro, Tanzania

^3^ Department of Food and Agricultural Sciences, Rovuma University, Cabo Delgado, Mozambique

^4^ Department of Animal Science, College of Agriculture, University of São Paulo, Avenida Padua Dias, 11, PO Box 9, Piracicaba, São Paulo, Brazil

^5^ Department of Veterinary Microbiology, Parasitology and Biotechnology, College of Veterinary Medicine and Biomedical Sciences, Sokoine University of Agriculture, PO Box 3019, Morogoro, Tanzania

***Correspondence:** [leonildo.viagem@sacids.org](mailto:leonildo.viagem@sacids.org) (LAV) and [gerald.misinzo@sacids.org](mailto:gerald.misinzo@sacids.org) (GM)

**S2 Table.** Complete database search strategies and the number of articles accessed in each database

| **Keyword** | **Results** | | | |
| --- | --- | --- | --- | --- |
|  | **Google Scholar** | **ScienceDirect** | **PubMed** | **Wiley Online Library** |
| “seaweed” OR “macroalgae” NOT “microalgae” AND “growth performance” AND “feed utilization” AND “tilapia” | 525 | 63 | 0 | 1,317 |
| “soybean” NOT "soybean fermented" AND “growth performance” AND “feed utilization” AND “tilapia” | 9 | 1,035 | 5 | 838 |
| “seaweed” OR “macroalgae” NOT "microalgae" AND “gut microbiota” OR “intestinal microbiota” AND “tilapia” OR “other fish” NOT "human" | 429 | 294 | 1,437 | 5,081 |
| “soybean” NOT "soybean fermented" AND “gut microbiota” OR “intestinal microbiota” AND “tilapia” OR “other fish” NOT "human" | 5 | 1,283 | 1,437 | 4,233 |
| **Total** | **968** | **2,675** | **2,879** | **11,469** |
