# Supplementary figures and images for: Effect of soybean and seaweed-based diets on growth performance, feed utilization, and gut microbiota of tilapia: A systematic review and meta-analysis
Source: PLoS One. 2024 Jul 24;19(7):e0293775. doi: 10.1371/journal.pone.0293775 (PMC11268637; doi:10.1371/journal.pone.0293775)

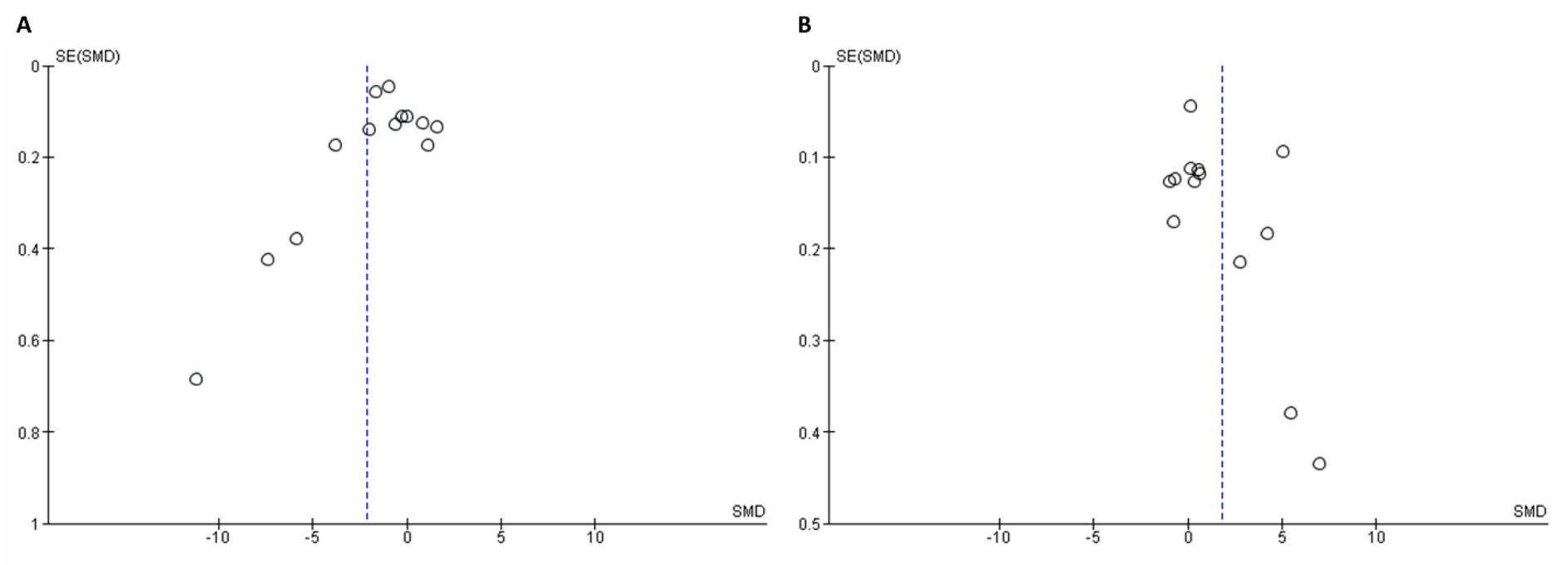

Supplement: S1 Fig — Publication bias represented by funnel plot of individual studies about the effect of soybean on SGR (A) and FCR (B). (TIF) [file pone.0293775.s004.tif]

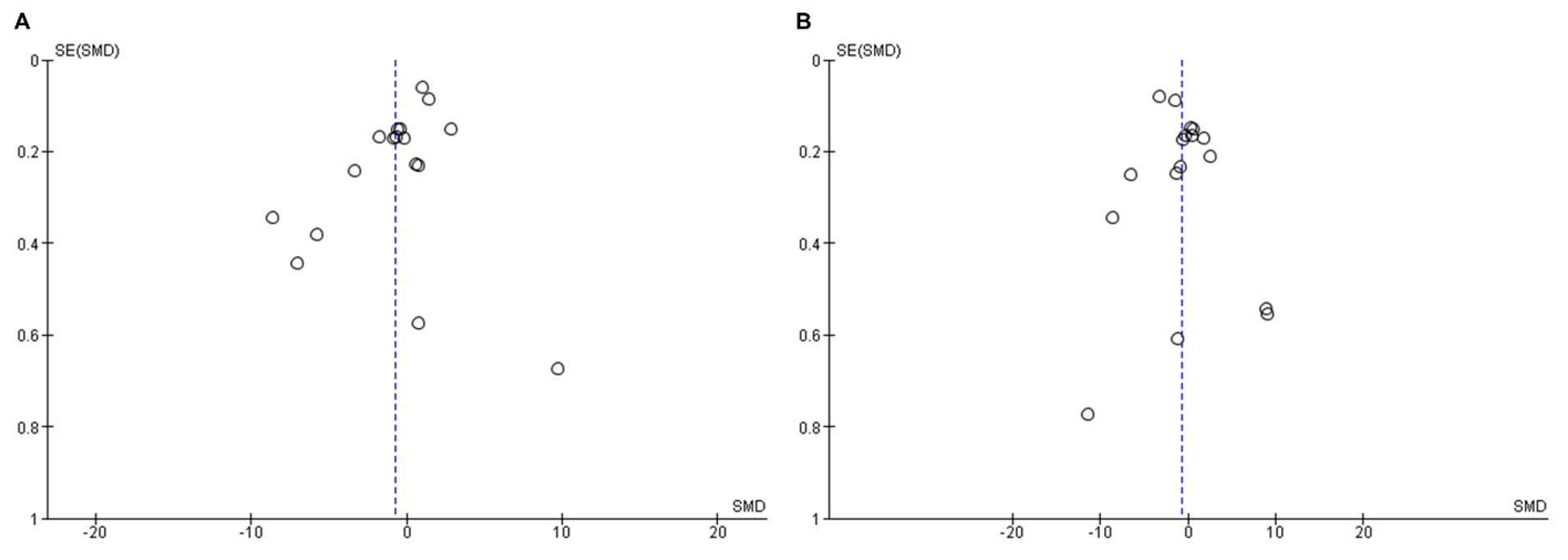

Supplement: S2 Fig — Publication bias represented by funnel plot of individual studies about the effect of seaweed on SGR (A) and FCR (B). (TIF) [file pone.0293775.s005.tif]
